# Reassessing Bipolar Cautery in Thyroidectomy: An Affordable Alternative to Advanced Energy Devices in Resource-Poor Settings

**DOI:** 10.7759/cureus.91085

**Published:** 2025-08-27

**Authors:** Jim Job, Somy Charuvila, Sona Vijay, Jithu P Skariah, Jasira Padinhare Madathil, Pradeep Chandran, Subramanian Ezhumalai

**Affiliations:** 1 General Surgery, Princess Royal University Hospital, Orpington, GBR; 2 General Surgery, South London General Surgery Training Programme, Health Education England (HEE), London, GBR; 3 General Surgery, Dr. Moopen's Wayanad Institute of Medical Sciences, Wayanad, IND; 4 Public Health, St. Mary's Hospital, Kottayam, IND; 5 General Surgery, Government Medical College, Thrissur, IND; 6 General Surgery, Government Medical College, Thiruvananthapuram, IND; 7 Surgery, Government Medical College, Thiruvananthapuram, IND

**Keywords:** bipolar forceps, hypocalcaemia, low- and middle-income countries, recurrent laryngeal nerve injury, thyroid surgery

## Abstract

Introduction

Thyroidectomy is one of the most commonly performed surgeries around the world. Historically, it was a high-mortality procedure. However, this is not the case today due to advances in surgical technique and instruments.

In particular, advanced energy devices such as LigaSure™ (Medtronic, Minneapolis, MN, USA) and Harmonic scalpel (Ethicon, Raritan, NJ, USA)* *have revolutionised thyroid surgery by enhancing precision and minimising blood loss. These devices are indispensable in most settings. However, in many low- and middle-income countries (LMICs), their high cost and limited availability pose significant barriers to their widespread adoption.

Standard bipolar forceps, on the other hand, is widely available in many LMIC settings and is significantly cheaper. In this study, we investigate the safety profile of using standard bipolar forceps in thyroid surgery. This study aimed to investigate the rates of the two most common complications post-thyroidectomy, namely, hypocalcaemia and recurrent laryngeal nerve (RLN) injury.

Methods

A prospective observational study of 178 patients undergoing total thyroidectomy for multinodular goitre was conducted at a single centre in Kerala, India. Postoperatively, all patients were evaluated for hypocalcaemia and RLN injury at six hours and then monthly for six months. Complications persisting at six months were deemed permanent. Any symptoms that resolved before six months were classified as temporary.

Results

The mean age was 40.8 years, 84/178 patients had symptoms lasting more than a year, and 156/178 patients had at least one nodule measuring ≥3 cm. Out of 178 patients, 118 were female and 60 were male.

Around 18.5% (33/178) of patients developed temporary hypocalcaemia. However, only one patient had permanent hypocalcaemia.

About 5.1% (9/178) of patients developed temporary RLN injury, and none of the patients had permanent RLN injury. One patient developed a wound infection, and the mortality rate was 0%.

Conclusion

This study outlines the outcomes of employing bipolar forceps for thyroid surgery, highlighting its potential as a safe and cost-effective substitute for advanced energy devices in low-resource settings.

## Introduction

Thyroid conditions are highly prevalent worldwide and particularly endemic in areas where iodine deficiency and environmental factors contribute to the burden of disease. Where medical therapy is inadequate, surgery offers definitive treatment. Historically, thyroid surgery was associated with high mortality and morbidity. However, the safety profile of thyroid surgery has improved with refinement in dissection techniques and implementation of tools such as intraoperative nerve monitoring, loupes, and advanced energy devices. 

The two most significant complications in relation to thyroid surgery are hoarseness due to recurrent laryngeal nerve (RLN) injury and hypocalcaemia (from inadvertent parathyroidectomy/injury). Effective haemostasis reduces the risk of RLN injury and hypocalcaemia as it improves intraoperative visibility, which is particularly pertinent in thyroid surgery where the operative field is limited. It is also important in reducing the risk of postoperative haematoma [1]. Advanced energy devices enable precise and effective haemostasis and have revolutionised the safety profile of thyroid surgery in the modern day [2]. However, the associated cost and limitations in procurement pose significant barriers to its adoption in many low-income settings. However, standard bipolar forceps are widely available, are significantly cheaper, and could be utilised as an alternative with careful dissection technique [3,4].

In addition to this, bipolar forceps also limit the heat dissipation to surrounding tissues which is a crucial factor to consider in thyroid surgery [2]. Heat injury can cause permanent damage to the RLN compared to traction injuries which are transient. This is because thermal energy can cause damage to the inner endoneurium, whereas the latter tends to mainly affect the outer layer of the nerve [5]. The jaw temperature, thermal spread, and cooling time should be taken into consideration while using advanced energy devices such as LigaSure™ (Medtronic, Minneapolis, MN, USA) and Harmonic scalpel (Ethicon, Raritan, NJ, USA), and there is a recommended 2-3 mm safety distance from the nerves. In comparison, the thermal spread for bipolar forceps is approximately 1 mm, and unlike other energy devices, bipolar forceps generate heat only at the inner contact surface of the jaws, while the external aspect remains cool, reducing the risk of unintended thermal injury [2]. 

It should also be emphasised that the effectiveness of any energy device is not only dependent on the device features but also influenced by the surgeon's experience and familiarity with the instrument [5]. Surgeons with high case volumes tend to have overall lower complication rates [6]. Hence, a surgeon's experience is crucial in the selection of energy devices. 

However, in the context of low-income settings, perhaps the most deciding factor in choosing an energy device is its associated cost and availability. In many low-income settings such as the centre where this research is conducted, while surgery is subsidised by the government or provided free of cost, the cost of the associated equipment is incurred by the patient. Thyroid surgery typically generates significant out-of-pocket costs even in developed nations [7]. 

In the local region where this study was conducted, the cost of a single-use advanced energy device (approximately 30,000 rupees) can be as high as 100 times the daily wage of an average labourer in India (300 rupees) [8]. 

Hence, it is important to evaluate the safety and efficacy of widely available cheaper alternatives to advanced energy devices. Standard bipolar forceps is cheaper (approximately 2000 rupees) and widely available in comparison to advanced energy devices such as LigaSure™ and Harmonic. 

The aim of this study was to analyse the rate of hypocalcaemia and RLN injury in patients undergoing total thyroidectomy with bipolar forceps dissection technique. Specifically, in this study, we distinguish between the rates of temporary and permanent hypocalcaemia and RLN injury. 

## Materials and methods

A prospective observational study of 178 consecutive patients was conducted at Government Medical College, Thiruvananthapuram, in Kerala, India, over a period of one year. 

Inclusion criteria

Patients undergoing total thyroidectomy between 20 and 80 years of age with a diagnosis of multinodular goitre were included in the study.

Exclusion criteria

Patients with a history of previous RLN pathology or parathyroid disorders or suspected or documented carcinoma of the thyroid or those who declined consent were excluded from the study.

Ethics

Ethical approval for this study was granted by the Human Ethics Committee of Government Medical College, Thiruvananthapuram (approval number: 06/17/2019/MCT). Written informed consent was obtained from all participants. Consent forms and information leaflets were provided in the local language (Malayalam). 

Preoperative evaluation

All patients underwent indirect laryngoscopy to assess their preoperative vocal cord function. A thorough history and physical examination were also carried out to identify symptoms related to parathyroid disorders or hypocalcaemia.

Serum calcium was measured in cases where features such as perioral numbness, seizures, Trousseau's sign, or Chvostek's sign were present. Serum calcium less than 2.20 mmol/L was considered diagnostic for hypocalcaemia.

Surgical procedure

Total thyroidectomy was performed under general anaesthesia using the standard capsular dissection technique. Standard reusable bipolar forceps were used for the dissection. It should be emphasised that no proprietary or specialised bipolar forceps were used. Specific equipment settings were not standardised, as these are adjusted intraoperatively according to tissue characteristics and surgeon preference.

All cases were carried out by faculty members of our department with a total of 15 surgeons involved, reflecting standard practice in our institution. All patients received standard anaesthesia care and perioperative care as per local guidelines.

Postoperative assessment for RLN injury

All patients were clinically evaluated six hours post-surgery for symptoms of hoarseness or stridor. If abnormalities were suspected, indirect laryngoscopy was performed immediately. Follow-up continued in the outpatient department on a monthly basis for six months. If abnormalities or symptoms arose during follow-up, indirect laryngoscopy was performed.

Complications persisting at six months were deemed permanent. Any symptoms that resolved before six months were classified as temporary.

Postoperative assessment for hypocalcaemia

Serum calcium was measured, and symptoms were closely monitored six hours post-surgery. Hypocalcaemia was suspected if perioral numbness, seizures, Trousseau's sign, and Chvostek's sign were present. Serum calcium less than 2.20 mmol/L was considered diagnostic for hypocalcaemia. All patients had serum calcium tested at six hours post-surgery. 

For patients with symptomatic or biochemical hypocalcaemia, calcium replacement was administered as per local guidelines. All patients were followed up monthly for six months. Hypocalcaemia was classified as temporary if it resolved within six months without continued supplementation and permanent if supplementation was still required at six months.

Other complications, such as seroma, wound infection, or haematoma, were also documented during follow-up.

Data collection and analysis

Relevant data points were extracted from patient records and entered into a Microsoft Excel spreadsheet (Microsoft Corp., Redmond, WA, USA) without patient-identifiable details. Data was then analysed by clinicians who were not involved in patient care.

Data was analysed using descriptive statistics. Categorical variables were expressed as frequencies and percentages and continuous variables as mean±standard deviation (SD) as appropriate. Comparisons between categorical variables were performed using the chi-squared test or Fisher's exact test, as applicable. All analyses were performed using Microsoft Excel. No adjustments were necessary for missing data as there were no patients who were lost to follow-up. 

## Results

Table 1 details the demographics of the 178 patients who underwent total thyroidectomy with bipolar cautery.

**Table 1:** Demographics of the patients included in this study

Patient demographics		n
Sex	Male	60
	Female	118
Age in years: 40.8±9.1 years (mean±SD)	≤30	22
	31-40	71
	41-50	65
	>50	20
Duration of symptoms	≤1 year	94
	>1 year	84
Largest nodule size: 3.4±1 cm (mean±SD)	<3 cm	22
	≥3 cm	156
Location of the largest nodule	Right side	101
	Left side	77
Socioeconomic demographics	Clerical/service jobs	86
	Vocationally trained jobs	16
	Daily wage labourers	8
	Homemakers	58
	Unemployed	10

About 66.3% of the patients were female (n=118). The mean age was 40.8 years, with a SD of 9.1 years. The majority of patients (76.4%) were in the 31-50-year age group. 

Around 47.2% of patients had symptoms of thyroid disease (swelling, dyspnoea, or dysphagia) for more than one year prior to surgery. All 178 patients had multinodular goitre as per the inclusion criteria, and in 87.6% of patients, their largest nodule measured ≥3 cm. The range of the largest nodule size in this cohort was 2-5 cm. The mean size of the largest nodule was 3.4 cm with a SD of 1 cm. The largest nodule was located on the right side in 56.7% of cases.

About 10% of patients' employment status reflects a poor socioeconomic background (daily wage labourers and unemployed). Around 32.6% of patients were classed as homemakers typically reflecting a single-income household. 

Postoperative complications

Only one patient developed a postoperative wound infection that was treated with oral antibiotics.

Other complications noted were RLN injury and hypocalcaemia, figures of which are outlined as follows. 

RLN Injury

Transient RLN injury occurred in 5.1% of patients (9/178). No patient experienced permanent RLN injury. All cases of RLN injury were identified in the immediate postoperative period and had resolved by the time of the first follow-up at one month post-op. At each subsequent month of the follow-up period of six months, there were no patients identified as having RLN injury.

There was no statistically significant difference in the rates of RLN injury in relation to nodule size, location of the nodule, duration of symptoms, or age or sex of the patient as seen in the figures in Table 2.

**Table 2:** Assessing clinical variables in relation to the risk of RLN injury. It should be noted that all cases of RLN injury were temporary and had resolved by the one-month post-op follow-up. RLN: recurrent laryngeal nerve

Patient/nodule characteristics	RLN status		n	P-value
	No RLN injury	Transient RLN injury	Total	
Nodule size				
<3 cm	19	3	22	Fisher's exact test (p=0.08)
≥3 cm	150	6	156	
Total	169	9	178	
Lateralisation of the largest nodule				
Right	96	5	101	Fisher's exact test (p=1)
Left	73	4	77	
Total	169	9	178	
Duration of symptoms				
≤1 year	92	2	94	Fisher's exact test (p=0.086)
>1 year	77	7	84	
Total	169	9	178	
Age (years)				
≤30	20	2	22	Chi-squared test (p=0.72)
31-40	67	4	71	
41-50	63	2	65	
>50	19	1	20	
Total	169	9	178	
Sex				
Male	57	3	60	Fisher's exact test (p=1)
Female	112	6	118	
Total	169	9	178	

Hypocalcaemia

Hypocalcaemia was observed in 34 patients (19.1%) in the immediate postoperative period. Of these, 33 (18.5%) had transient symptoms, while one patient (0.6%) had persistent biochemical hypocalcaemia at six months. The majority of patients with hypocalcaemia only had mild reductions in serum calcium, with no patient requiring intravenous calcium supplementation.

**Figure 1:**
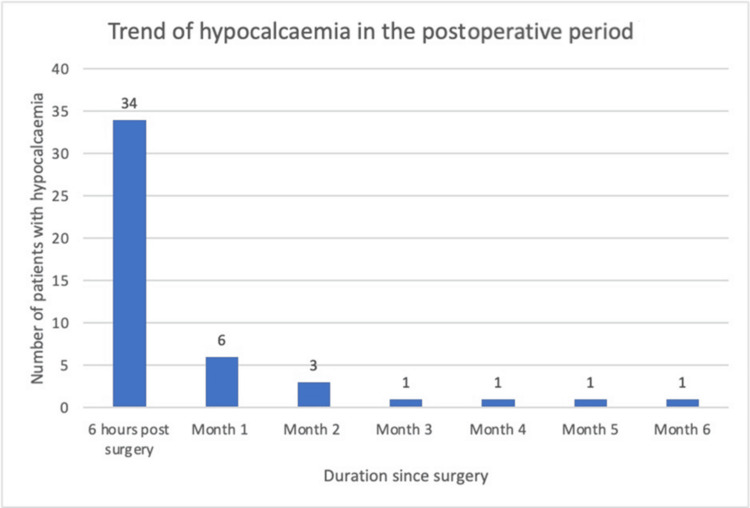
Trend of the number of patients with hypocalcaemia in the postoperative period.

The trend of hypocalcaemia can be noted in Figure 1 where the number of patients with hypocalcaemia decreases over time.

The association between hypocalcaemia in relation to age, sex, nodule size, and duration of symptoms were also explored as seen in Table 3. The only statistically significant association was seen in cases where the largest nodule size was ≥3 cm, which was seen to increase the risk of hypocalcaemia. 

**Table 3:** Assessing clinical variables in relation to the risk of hypocalcaemia. This table includes the details of all patients noted to have hypocalcaemia (temporary and permanent).

Patient/nodule characteristics	Calcium status		n	P-value
	Normal calcium	Hypocalcaemia	Total	
Nodule size				
<3 cm	22	0	22	Fisher's exact test (p=0.009)
≥3 cm	122	34	156	
Total	144	34	178	
Duration of symptoms				
≤1 year	78	16	94	Fisher's exact test (p=0.537)
>1 year	66	18	84	
Total	144	34	178	
Age (years)				
≤30	16	6	22	Chi-squared test (p=0.136)
31-40	60	11	71	
41-50	55	10	65	
>50	13	7	20	
Total	144	34	178	
Sex				
Male	49	11	60	Fisher's exact test (p=1)
Female	95	23	118	
Total	144	34	178	

## Discussion

In this prospective observational study evaluating the use of bipolar forceps dissection in total thyroidectomy, the overall complication rates were low and comparable with those reported in the literature. Temporary hypocalcaemia occurred in 18.5% (33/178) of patients, while permanent hypocalcaemia was rare (0.6%, 1/178). Temporary RLN injury was observed in 5.1% of cases, with no instances of permanent RLN injury.

With the emergence of advanced energy devices, few papers in the literature focus on the use of bipolar forceps as a single modality for thyroidectomy. However, in low- and middle-income countries (LMICs) where resources are constrained, complication rates for standard bipolar forceps have been compared to the conventional clamp and tie technique. 

Prakash et al. in India compared bipolar forceps to suture ligation technique and found that the rates of hypocalcaemia and RLN injury were comparatively reduced with bipolar forceps dissection [9]. 

Das et al. also carried out a study of 130 patients who underwent total thyroidectomy using bipolar forceps in a rural tertiary centre in India and had comparable rates of permanent RLN injury (1.5%) and permanent hypocalcaemia (2.3%) to our study [10]. 

Vasuki et al. found that the use of bipolar forceps in thyroidectomy was associated with reduced operative blood loss when compared to the clamp and tie technique in a cohort of 50 patients [11]. 

Fewer drain insertions and early patient discharge were also benefits associated with bipolar forceps when compared to the conventional clamp and tie technique [12]. The use of bipolar forceps has also been compared to other dissection modalities. A blinded randomised study of 45 patients in Egypt compared the outcomes of bipolar forceps, Harmonic scalpel, and conventional technique. Here, there was no significant difference in the rates of hypocalcaemia and RLN injury. However, they found that patients in the bipolar forceps and Harmonic scalpel groups had significantly less blood loss and had shorter operative time when compared to the conventional technique [13]. 

Similarly, a study by Su et al. compared postoperative outcomes for bipolar forceps, Harmonic scalpel, and conventional technique (clamp and tie) and found that Harmonic scalpel and bipolar forceps were superior to the conventional technique [14]. However, they also noted that bipolar forceps had a significantly lower rate of temporary RLN injury when compared to the Harmonic scalpel group. This could be due to the lower thermal spread associated with bipolar forceps. In this study of 527 patients, no patients developed permanent RLN injury or permanent hypocalcaemia. 

A meta-analysis of 115 studies quoted that the median incidence of temporary hypocalcaemia and permanent hypocalcaemia was 27% and 1%, respectively [15]. Similarly, a systematic review of 63 studies quoted that the rate of temporary hypocalcaemia was 12.5% and permanent was 2.2% [16]. The hypocalcaemia rates in this study are comparable/lower at 18.5% (temporary) and 0.6% (permanent).

The incidence of RLN injury post-thyroidectomy also greatly varies in the literature with figures ranging from 0.4% to 7.2% for temporary injury and from 0% to 5.2% for permanent injury [17]. Studies have shown that the usage of intraoperative nerve monitoring reduces the risk of RLN injury [17,18]. In this study, the rate of temporary RLN injury was 5.1%, and no patients developed permanent RLN injury. It should be noted that the rate of RLN injury in this study is comparable/better despite the absence of intraoperative nerve monitoring in this resource-limited setting.

In addition to assessing the overall rates of hypocalcaemia and RLN injury, we also explored associations between clinical variables such as nodule size, duration of symptoms, and laterality. This was done to have a better understanding of the patient cohort and their risk profile. 

Patients with their largest nodule size ≥3 cm had statistically significantly higher incidence of transient hypocalcaemia (p=0.009). This is unsurprising, as larger nodules make the dissection challenging and can increase the risk of inadvertent parathyroid devascularisation or excision.

As seen in Table 1, the patient demographics reflect increased case complexity. For instance, 87.6% of the patients in our study had their largest nodule size ≥3 cm. Similarly, a delayed presentation could also correspond to more technically demanding surgery owing to nodule size and degree of fibrosis. Here, 47.2% of patients presented with their multinodular goitre more than a year after developing symptoms. In addition to this, 56.7% of the patients had their largest nodule on the right side. The RLN has a more variable and superficial course on the right side and theoretically poses an increased risk of RLN injury compared to the left RLN. 

Despite a complex cohort, and standard bipolar forceps being the only energy device, the overall complication rates of hypocalcaemia and RLN injury in this study are comparable/better compared to some of the aforementioned studies. This is particularly relevant in a low-income setting where the patient cohort can present late with larger nodules and are unable to afford the cost of advanced energy devices. 

In this study, 10% of patients were daily wage labourers or unemployed. As mentioned in the Introduction section, the cost of a single-use advanced energy device (approximately 30,000 rupees) can be as high as 100 times the daily wage of an average labourer in India (300 rupees) [8]. Additionally, 32.5% of patients were classed as homemakers typically reflecting a single-income household. Affordability is a key factor for consideration in such settings where patients incur out-of-pocket expenses and can experience catastrophic costs as a result of having surgery.

While other studies in the literature also evaluate the use of bipolar forceps in thyroidectomy, this study adds a unique perspective by emphasising the delivery of safe, affordable surgery in low-resource settings. We highlight the cost of equipment in relation to daily wage earnings, bringing to light the financial accessibility of this technique for patients who cannot afford high-cost energy devices.

Furthermore, our study includes a thorough six-month follow-up using objective assessments, including laryngoscopy and serum calcium measurements, with no patients lost to follow-up. The cohort comprised a complex case mix, with a significant proportion presenting with large nodules and delayed symptom duration, and we provide detailed socioeconomic data for our patient population. Together, these elements underscore the feasibility, safety, and cost-effectiveness of bipolar forceps thyroidectomy, offering insights that are relevant not only to resource-limited settings but also to publicly funded healthcare systems, where cost-conscious surgical approaches are increasingly important.

It is also important to acknowledge the limitations of this study. The absence of a control group is perhaps the biggest limitation. However, in this centre, surgeons have extensive expertise using bipolar forceps, and it has been the standard of care in recent years. Moreover, other studies in the literature as mentioned previously have already demonstrated that bipolar forceps have a lower complication profile compared to the conventional technique. In this study, the aim was to demonstrate the safety profile and feasibility of using bipolar forceps alone as a modality of dissection in the absence of advanced energy devices. Another limitation is that this is a single-centre study. 

## Conclusions

Despite our study limitations, our data highlights the suitability of bipolar cautery as a cost-effective and dependable option for thyroid surgery in similar contexts. In the future, multicentre studies in similar clinical settings could be conducted to obtain more robust large-scale data on further variables such as operative time and blood loss and follow-up patients for a longer duration. This can assess if the findings of this study can be applied in contexts where the case mix (cancer vs benign) and surgical expertise vary. 
